# Standards of practice in empirical bioethics research: towards a consensus

**DOI:** 10.1186/s12910-018-0304-3

**Published:** 2018-07-10

**Authors:** Jonathan Ives, Michael Dunn, Bert Molewijk, Jan Schildmann, Kristine Bærøe, Lucy Frith, Richard Huxtable, Elleke Landeweer, Marcel Mertz, Veerle Provoost, Annette Rid, Sabine Salloch, Mark Sheehan, Daniel Strech, Martine de Vries, Guy Widdershoven

**Affiliations:** 1 University of Bristol, Bristol, UK; 2 University of Oxford, Oxford, UK; 3 VU Medical Centre, Amsterdam, Netherlands; 4 Martin Luther University Halle-Wittenberg, Halle, Germany; 5 University of Bergen, Bergen, Norway; 6 Universty of Liverpool, Liverpool, UK; 7 University of Groningen, Groningen, Netherlands; 8 Hannover Medical School, Hannover, Germany; 9 Ghent University, Ghent, Belgium; 10 Kings College London, London, UK; 11 Greifswald University, Greifswald, Germany; 12 Leiden University Medical Centre, Leiden, Netherlands; 13 Berlin Institute of Health, Berlin, Germany

**Keywords:** Empirical bioethics, Methods, Methodology, Standards, Consensus

## Abstract

**Background:**

This paper reports the process and outcome of a consensus finding project, which began with a meeting at the Brocher Foundation in May 2015. The project sought to generate and reach consensus on standards of practice for Empirical Bioethics research. The project involved 16 academics from 5 different European Countries, with a range of disciplinary backgrounds.

**Methods:**

The consensus process used a modified Delphi approach.

**Results:**

Consensus was reached on 15 standards of practice, organised into 6 domains of research practice (Aims, Questions, Integration, Conduct of Empirical Work, Conduct of Normative Work; Training & Expertise).

**Conclusions:**

Through articulating these standards we outline a position that encourages responses, and through those responses we will be able to identify points of agreement and contestation that will drive the conversation forward. In that vein, we would encourage researchers, funders and journals to engage with what we have proposed, and respond to us, so that our community of practice of empirical bioethics research can develop and evolve further.

## Background

Empirical bioethics (EB) is a broad term that has been used to capture a range of different research activities [1]. This paper focusses on a particular approach that has been taken in predominantly European literature that frames empirical bioethics as an interdisciplinary activity in which empirical social scientific analysis is integrated with ethical analysis in order to draw normative conclusions. It is exciting, according to Ives et al. [1], because it is a field that potentially “promises a great deal”, but also frustrating “because the emerging field threatens to be so multifarious and vague that making sense of it is a challenge for even the most seasoned researcher” (ix). Much ink has been spilled in recent years either extolling or critiquing the rise of the ‘empirical turn’ in bioethics [1–3]. Whilst some commentators debate whether or not EB is a good or necessary thing [4, 5] others have articulated methods and methodologies for conducting empirical bioethics research and used them [6–20].

The challenges of this endeavor are by now well-known and well-rehearsed, and centre on: ontological^1^ and epistemological^2^ questions connected to how an empirical ‘is’ can inform a normative ‘ought’ claim; on disciplinary questions about the nature and core characteristics of the field itself; and on methodological questions regarding how to integrate the empirical and the normative part of EB. These challenges notwithstanding, or perhaps just because they are so contentious and difficult to overcome, numerous approaches to conducting such work have been described. In a recent systematic review, Davies et al. [21] identified 32 distinct EB methodologies that try somehow to ‘integrate’ the empirical and the normative. The authors of that review also warned that due to the heterogeneity of approaches it is difficult to present, defend or critically assess a piece of work in EB because:

‘The shortcuts that can be taken when explaining and justifying work undertaken within clear disciplinary silos are not available to empirical bioethics. There is no standard approach to cite, there is no accepted methodology or set of methods to fall back on, and the process of offering justification for every methodological choice from first principles takes a lot of space, which is rarely available’. (p12).

Davies et al. went on to suggest that a way forward may be to try to find some sort of consensus within the academy on what constitutes good work of this kind:

One way forward may be to attempt to find some level of consensus on what is required of an empirical bioethics methodology, and what standards we might use to assess the quality of work proposed and/or undertaken under this broad umbrella. Establishing a consensus that outlines areas of agreement may be able to provide some external and relatively concrete validation for at least some kinds of work. Such areas of agreement might include, for example: what assumptions one may legitimately make, and whether the theoretical assumptions behind one’s approach need to be stated, explained or fully justified (from first principles). (p12).

We believe that there are potential benefits from attempting to find some level of consensus regarding what EB is and what we conceive as a minimum methodological quality.

A central benefit of having some agreement on what is required of an EB study (in design, conduct and reporting) is that it is likely to help cement EB as a distinct ‘community of practice’ [22], with its own specific methodological norms and aims. Such a community of practice may help those engaging with it to conduct EB and to locate themselves in the academy. Having agreed and accepted standards may also help the community of practice to ensure and improve the quality of work, provide a springboard for future methodological developments, and be useful in training (PhD) researchers. Those who plan EB can then pro-actively reflect upon the design and quality of their research. Conversely, if no consensus is possible, that might suggest that EB may not be tenable as a community of practice.

Additional benefits of trying to establish standards of practice include:

1. It is potentially beneficial to researchers seeking funding, who may be able to point to areas of agreement to support certain methodological choices and decisions, or similarly to challenge assertions about inappropriate methodological choices that are in fact moot.
2. It is similarly useful for researchers seeking publication to have a clear idea of what is expected in terms of reporting, which can guide their writing.
3. It is likely to be beneficial to journals, funders, and the peer reviewers they draw on, to be able to point to an agreed set of practice standards for empirical bioethics, which can be used to inform and guide quality assessment.
4. It is likely to be beneficial for those who train or teach students and researchers in the field of EB.

These benefits are particularly relevant in a field where interdisciplinarity in some shape or form is ubiquitous, and in which the challenges of answering an individual research question might require deviations from standard disciplinary approaches. Early pioneers of this kind of work recognized that standard approaches to data collection, for example, may have to be altered in order to generate data that could help them meet their particular aims, with focus groups being turned into something more interrogative and challenging - more ‘akin to a philosophy seminar’ - than they might normally be. [23] This kind of methodological innovation is to be expected in a developing interdisciplinary field but, as Frith and Draper [22] have pointed out, it presents challenges when engaging with a disciplinary audience, which might tend to appraise the work according to its own norms.

Given these potential benefits, the call for the development of research practice standards in EB has already begun to be answered. In an important recent contribution, members of the empirical ethics working group of the Academy of Ethics in Medicine in Germany have outlined what they describe as a “road map for quality criteria” in empirical ethics research [24]. Motivated by the same concerns about the broad variety of methodologies adopted within interdisciplinary inquiry of this kind, these authors categorised quality criteria on the basis of the i) primary research question, ii) theoretical framework and methods, iii) relevance, iv) interdisciplinary research practice, and v) research ethics and scientific ethos. Drawing on quality criteria for empirical research in the social sciences, and for philosophical inquiry in practical ethics, they identified formal norms, cognitive norms, and ethical norms that gave shape to their ‘road map’ of basic criteria for good quality interdisciplinary empirical ethics research. Formal norms refer to those practices associated with research writing; cognitive norms to those practices associated with general methodological commitments and the deployment of appropriate forms of analysis; and ethical norms to those practices associated with the moral conduct of the research activities.

Mertz et al.’s [24] proposal is focused on adopting standards drawn from other areas of inquiry to provoke empirical ethics researchers to reflect carefully on the choices they make in their studies. Whilst this road map commits researchers to certain criteria, it is unclear – as the authors themselves acknowledge – whether this starting point provides precise enough guidance to be useful to the various audiences that we believe could benefit from clarifications of this kind. It also looks to be important to undertake this standard-development exercise in ways that involve, and thus can command broad agreement within, the varied communities of EB researchers themselves.

Mindful of the risks involved in this kind of activity, this paper reports an attempt to develop a consensus amongst some European practitioners of EB about standards of practice and reporting in EB research. We take the work of Mertz et al. as a starting point, and seek to further develop this body of work by undertaking a transparent and rigorous consensus seeking process, utilizing the perspectives of a range of expert scholars with different disciplinary expertise, purposively selected to represent different and putatively opposing EB positions. We sought to generate, as far as possible, a set of agreed standards that are precise enough to provide concrete guidance for (a) identifying the core characteristics of EB (b) planning EB (c) conducting EB and (d) reporting EB. In what follows, we describe the process and the consensus that was reached, and we discuss the implications that we see for the on-going development of a field that is committed to these standards.

## Methods and results

The lead authors of this paper (JI, MD, BM, JS) applied for funding from the Brocher Foundation to hold a 2.5 day meeting (May 2015) in which an attempt would be made to generate standards of practice and reach consensus. We utilised an adapted Delphi process, which is a recognised and well established technique for reaching consensus and is amenable to modification to suit specific aims [25]. The Delphi method, broadly speaking, is a structured consensus finding process, which typically involves administering questionnaires to expert participants in an iterative series of rounds. After each round, feedback is provided to participants that summarizes the views of the group, suggests a common position, and then seeks further input. According to Hsu & Sandford [26].

“The feedback process allows and encourages the selected Delphi participants to reassess their initial judgments about the information provided in previous iterations. Thus, in a Delphi study, the results of previous iterations regarding specific statements and/or items can change or be modified by individual panel members in later iterations based on their ability to review and assess the comments and feedback provided by the other Delphi panelists.” (p2).

Typically this process maintains the anonymity of participants; the putative advantage of which is that it frees participants from pressure to conform to the group view. Our adaptation of this method differed in two important ways. First we did not seek feedback through multiple rounds of questionnaires, but through group discussion. Second, and as a consequence of this, participants were clearly identifiable to one another. This adaptation was justified, we felt, by the pressing need to have clear and robust direct verbal communication through discussion that allowed disagreements to be aired and mutually understood, and which facilitated a sense of the group having a clearly defined and shared goal. Given the linguistic and conceptual diversity of the field, and given that the majority of the groups were working in a second language, we felt it would be very problematic to use questionnaires – which rely on an assumption of clarity and shared understanding. Given that we could not take shared language and conceptual understanding for granted, we needed a process that allowed participants to immediately respond and ask for clarification as and when an ambiguous or controversial issue was raised. Rather than having a series of iterative questionnaires, we therefore had a series of iterative discussion groups, with regular summary and feedback sessions (acting as a ‘member check’), in which we iteratively developed a set of agreed ‘domains’ (categories of research activity in which standards might be required), then standards within those domains, and finally concrete formulations of standards within those domains.

One potential weakness introduced by the discussion group modification is that participants may have felt unable to challenge what they saw as the prevailing group view. This is always a risk in any group process, but it was mitigated in various ways. First, the round 1 process (detailed below) in which standards were developed was carried out in small groups, which changed on each rotation, meaning that there was no single group view that could dominate in the early stages. Second, the final stage of the process was carried out via online voting and feedback, and so all participants had the opportunity to agree or disagree without being observed by the rest of the group and risking being seen as ‘deviant’. Finally, in our opinion, during the discussion sessions we saw no indication that any participants were reluctant to speak or felt unable to offer a dissenting opinion – quite the opposite, in fact, with discussion at times becoming quite heated. Overall, given the benefits of the modification (as described above), and given the steps that were taken to mitigate the potential risks, we are confident that the process was robust.

We have chosen here to present the methods and results together in a single narrative, which allows us to better, and more efficiently, articulate our thinking at each stage. Whilst we present our process in some detail, we feel that this is necessary in order to demonstrate transparency and rigor. In presenting the process and results together we do risk some ambiguity about the focus of the paper. We stress that our primary goal is to present and discuss the results of our consensus, and the detailed presentation of our method is a means to achieving that.

### Participant selection

Space and funding was available for up to 20 participants at the meeting, limited to those residing in Europe. In selecting participants to invite, a number of factors were considered and had to be balanced. Key considerations were: previous contributions to and standing in the field; gender balance; avoiding over-representation of a single country; ensuring ‘disciplinary’ diversity, ensuring diversity of known ‘meta-ethical’^3^ and methodological positions. This level of diversity was sought to try to ensure that we did not create an ‘echo chamber’ in which a group of like-minded people could easily agree.

Achieving a balance within the imposed limits on numbers meant that not everyone who has contributed significantly to the field could be invited, and so we opted for a mix of participants who could represent the various kinds of interests that operate within the field. In light of this, we make no claims to have achieved a universal consensus – hence our title of ‘*towards* a consensus’.

Potential participants were contacted individually and asked to participate, and if an invited participant could not attend, we sought an alternative who would add similar diversity to the group. No-one approached refused to take part, but some were unable to attend the consensus meeting and therefore could not take part in the project. In total, and including the meeting organizers, 30 people were approached to participate.

Ultimately, the consensus group comprised 16 members from 5 different countries (see Table 1).

**Table 1:** Delphi members

Name (in alphabetical order)	Country	Disciplinary orientation
Bærøe, Kristine	Norway	Bioethics; Philosophy
De Vries, Martine	Netherlands	Bioethics; Medicine
Dunn, Michael (Chair)	United Kingdom	Bioethics; Social Science
Frith, Lucy	United Kingdom	Bioethics; Social Science; Philosophy
Huxtable, Richard	United Kingdom	Medical law; Bioethics
Ives, Jonathan (Chair)	United Kingdom	Bioethics; Philosophy; Social Science
Landeweer, Elleke	Netherlands/Norway	Bioethics; Philosophy; Social Science
Mertz, Marcel	Germany	Philosophy; Social Science; Bioethics
Molewijk, Bert (Chair)	Netherlands/Norway	Clinical ethics; Social Science
Provoost, Veerle	Belgium	Bioethics; Social Science; Methodology
Rid, Annette	United Kingdom	Medicine; Philosophy; Bioethics
Salloch, Sabine	Germany	Medicine; Philosophy; Bioethics
Schildmann, Jan (Chair)	Germany	Medicine; Philosophy; Bioethics
Sheehan, Mark	United Kingdom	Bioethics; Philosophy
Strech, Daniel	Germany	Bioethics; Health Policy Analysis; Meta-Research
Widdershoven, Guy	Netherlands	Bioethics; Philosophy; Social Science

As with any consensus process, the consensus that is ultimately produced cannot be representative or generalized, and is limited to the group within which the consensus was reached. The fact that this group was limited to European researchers immediately implies that the consensus reached is a product of a broadly European perspective. Further, this process did not (and could not) include all European empirical bioethics researchers, and so the consensus cannot be considered to be speaking for all empirical bioethics researchers in Europe.

Perhaps the most salient limitation imposed by the participants selected is that the kind of empirical bioethics that has demanded the most attention in Europe is more focused on the process of integrating the theoretical and the empirical than elsewhere in the world. As Ives et al. [1] note in the preface to their book.

“This focus on integration is significant because it demarcates our account of empirical bioethics from other research strategies that are commonly included under this term. We take it that there are a number of other ways in which empirical research can be put to work in bioethic. The empirical identification of ethical issues in practice, the empirical substantiation of practical moral arguments and the empirical evaluation of the implementation of ethical arguments/interventions into practice are commonly included in ‘broader church’ typologies of empirical bioethics” (px).

Of course, it would be a mistake to assume that there is a single European perspective, and our selection strategy (as outlined above) sought to select for diversity in perspectives. Whilst all were able to agree at the outset that the focus of the consensus would be on an empirical bioethics that integrates the empirical and the normative, there was still a great deal of diversity within the group, which is demonstrated in the results and discussion below.

### Pre-meeting consultation via email

The first stage was a pre-meeting consultation process, in which the aims and scope of the meeting were made clear by the four organizers (JI, MD, BM, and JS). Participants were asked by email to consider, as a starting point, a prepared list of suggested domains under which standards might be needed and some associated questions. These domains were identified by the lead authors through a non-systematic review of the empirical bioethics literature. The instruction to participants was as follows:

*This pre-workshop exercise is intended to begin to map out the ‘domains’ under which agreed standards may be possible/required, and will be used as the starting point for the workshop on the 11th May. On the first day of the workshop, we will refine these ‘domains’ and limit further discussion to a smaller sub-sample that we identify, as a group, as being the most significant.*

*We have designed the workshop in this way due to the wide range of research strategies adopted in the field, which may presume a range of different and mutually exclusive kinds of theoretical and methodological commitments. We seek to explore how standards and quality may be assessed, rather than how the research ought to be done.*

*In order to make progress in the workshop on research standards, we intend to limit discussions to focus on a particular account of empirical bioethics. This is the account that we adopted to obtain funding for the meeting and, we hope, is minimal enough to be accepted by all those attending the workshop: “empirical bioethics is an approach to bioethics in which empirical social scientific analysis is integrated with ethical analysis in order to draw normative conclusions*.”

Participants were then asked to begin to think about, and send comments on, the kind of topics and questions we anticipated engaging with. This was intended not to direct the content of such statements, but to collect a broad range of various topics and questions that might be relevant for the standards we wanted to develop and to encourage participants to begin to think about the nature and form of what we might seek consensus on. The list of 11 domains and questions/topics that was sent to participants is presented in Table 2 below.

**Table 2:** List of domains and sample questions for the pre-meeting exercise

Domain	Example question/topic
Aims	What are appropriate aims for EB research?
Questions	What kinds of questions can and should EB research answer?
Research ethics	What distinctive ethical considerations should be foregrounded in the conduct of empirical bioethics research?
Researcher characteristics/background	What kinds of researchers ought (not) to be undertaking EB research?
Researcher training	What kinds of trainings/skills/experience ought an EB researcher to have?
Nature of research team	Can/should EB research be practiced by a research team or by an individual, or by either?
Methodology	How precisely should social scientific and ethical inquiry be integrated in order to draw normative conclusions?
Methods	What are legitimate methods to use in EB research?
Nature/scope of normative justification	What level of justification/support/ explication of normative argument are required in EB research?
Conclusions	What kinds of conclusions are appropriate for EB research?
Publishing	How should methodological approaches and methods be documented in papers submitted for academic publication?

Participants were asked to send feedback on the following questions:

1. Are any domains missing? If so, which?
2. Should any domains be removed? If so, why?
3. Are any of the domains listed problematic or very complexes? If so, why?
4. Can you think of any important example questions/topics to add under any domain?

### The 2.5 day meeting

#### Item generation

Following a brief introductory talk from the organizers at the start of the 2.5 day meeting, which reinforced the aims (i.e. reaching consensus) and set some ground rules (e.g. mutual respect, agreement to focus on reaching consensus), the feedback from the pre-meeting consultation was presented, as raw data. This was done with the aim of orienting the group to the kinds of concerns and issues likely to come up, and with a view to helping everyone start to think about the topic and get a sense of how their colleagues were thinking.

What then followed was a series of 6 rounds that developed and sought agreement on ‘domains’ and ‘standards’. Domains were defined as ‘organizational categories that encapsulate a specific and meaningful part of the research process’ and standards were defined as ‘prescriptive statements about the conduct of work within a domain’.

##### Round 1 (day 1) – Identifying domains

The aim of this round was to look specifically at all the domains that had been proposed in the pre-meeting consultation and propose changes (i.e. add or delete domains, redefine domains, etc.). Participants were randomly split into four groups, facilitated by one of the meeting chairs (JI, MD, BM, JS), and each group considered all the domains. After this round, each group came up with their own list of all relevant domains under which standards were thought to be required.

##### Round 2 (day 1) – Agreeing domains

The four groups were then recombined, and a member of each of the four groups presented the domains developed during their discussions, highlighting areas of agreement, disagreement and uncertainty within their group. All domains were recorded, and the meeting chairs began to group similar domains together as they were presented, making note of areas of similarity and disagreement between the different groups’ lists. Some domains were clearly endorsed by all groups, and a discussion about nomenclature was held to agree on the correct terms to use. Discussions were also held about whether certain domains were the same and needed combining, or were in fact distinct. By the end of round 2, there was a list of 10 domains (see Table 3 below).

**Table 3:** List of agreed domains after phase 2

	Aims
1	Questions
2	Integration
3	Conclusions
4	Conduct of empirical work
5	Conduct of normative work
6	Training
7	Output
8	Research ethics and integrity
9	Multi−/inter-disciplinarity

##### Round 3 (day 2) – Identifying domain standards

Participants were again randomly split across 4 four discussion stations, each facilitated by one of the meeting chairs (JI, MD, BM, JS). Each station was allocated a number of the previously agreed domains, the number being determined by the anticipated complexity of identifying standards for it.

Each station worked for 30 min, and then the groups were split and participants were rotated into another station whilst the facilitator remained. Each participant was rotated into each station, working with different people for 30 min, before returning to their original station for the final rotation. Each proceeding group at each station worked on and developed the work of the preceding group, with the facilitator remaining throughout to explain the reasoning behind the previous group’s decisions and ensure that the process and decisions were recorded so that they could be presented back to the wider group. Each group working at each station identified and revised standards for each domain they were working with, which included both principled discussions about what standards, should be used and how each standard ought to be articulated. At the end of this round, each domain had a set of draft standards, which had been co-developed by all participants over the course of 2.5 h.

##### Round 4 (day 2) – Ranking and formulating domain standards in small groups

The aim of this round was to spend time revising and developing clear and precise articulations of each draft standard. Participants were given a choice about which domain they had most interest in or felt they had most to contribute to, and they naturally split themselves into equal size groups based on interest. Each group spent 2 h talking through all standards under each domain, and working on the precise formulation of each. Groups were told they could decide to reject a standard if they felt it was not possible to agree on a sufficiently specific form of words. Another reason for rejecting a standard, or a whole domain, at this stage was if the group felt that it was not sufficiently useful or fit for purpose.

##### Round 5 (day 2–3) - ranking and formulating domain standards in a large group

After a break, the whole group was reconvened, and each facilitator took the group through each standard’s formulation and explained the reasons for the proposed wording. Each individual standard was then debated, allowing as much time as necessary to either achieve an uncontested formulation or to be certain that more work was needed. Given this, the planned timetable was adjusted, and it was agreed that round 5 would continue into the final day, and the consensus vote would take place post-meeting. By the end of the 3rd day, there were proposals to reject some domains entirely, and there were still some standards that all agreed were required but for which no wording had been proposed that could be put to the vote.

At this point, it was agreed by the whole group that the meeting organizers would take all comments that had been made (and recorded in notes) and use those to propose a set of domains, standards and formulations that reflected the work done so far and which tried to take into account as many of the points and concerns raised as possible. These would then be circulated post-meeting and voted on, with further refinements made as needed according to a standard Delphi method. It was also proposed and unanimously agreed at this point that for consensus to be reached at least 80% of participants must endorse a standard. For a group of 16, this meant that 13 people had to endorse a standard in order to reach consensus.

### Post-meeting online rounds

#### Round 6a (post meeting) – First vote on domains and standards

Post-meeting, the organizers developed a set of domains and standards that seemed to best reflect the plenary group discussions, and circulated these in an online survey to the meeting participants. Table 4 presents the list of domains and standards that was circulated, as well as the domains that were not included and the reason (taken from the discussion in round 5) for non-inclusion.

**Table 4:** List of domains and standards circulated (and rejected) following discussion in round 5

Domain	Round 5 standards included	Reason for rejection
Aims	Empirical bioethics research should address a normative issue that is oriented towards practice	N/A
Aims	Empirical Bioethics research should integrate empirical methods with ethical arguments in order to address this normative issue	N/A
Questions	Empirical bioethics researchers ought to be explicit about how the research question(s) asked address(es) the normative issue identified in the aims	N/A
Integration	The theoretical position on integration (i.e. the theoretical views on how the empirical and the normative are related) should be made clear and explicit	N/A
Integration	The method of integration should be explained and justified, including details of what is integrated with what, how and by whom	N/A
Integration	There should be transparency, consistency and rigor in the execution and reporting of the integrating analysis	N/A
Conduct of empirical work	Empirical bioethics research ought to attend to the rigorous implementation of empirical methods, and import accepted standards of conduct from appropriate research paradigms	N/A
Conduct of empirical work	Empirical bioethics research should, if and where necessary, develop and amend empirical methods to facilitate collection of the data required to meet the aims of the research; but deviation from accepted disciplinary standards and practices ought to be acknowledged and justified	N/A
Conduct of empirical work	Empirical bioethics research should reflect on and justify the appropriateness and fit of the chosen empirical methods in relation to (a) the normative aims (b) the stated approach to integration	N/A
Conduct of empirical work	Empirical bioethics research should consider and reflect on the implicit ethical and epistemological assumptions of the chosen empirical method	N/A
Conduct of normative work	In empirical bioethics research there should be thorough delineation of the ethical issue(s), paying attention to, and locating them within, the relevant disciplinary literature	N/A
Conduct of normative work	In empirical bioethics research there should be an explicit and robust ethical argument, where argument is understood as an explicit attempt to convince X to adopt position Y with the use of reasons	N/A
Training and expertise	The empirical bioethics researcher, or the research team as a whole, should possess competence in ethical inquiry, empirical inquiry and methods of integration	N/A
Training and expertise	The empirical bioethics researcher(s) should have at least a basic knowledge of bioethics, and an understanding of whatever aspects of other disciplines or fields they are engaged with	N/A
Training and expertise	Provision should be made for ensuring that any team members can acquire or enhance competence in empirical bioethics research	N/A
Conclusions	None	This was not included as a domain because formulations of standards under it were (a) too prescriptive about the kind of research that could be undertaken or (b) those that were not too prescriptive simply repeated points already captured in standards within the ‘aims’ and ‘questions’ domains.
Training	None	This was not included as a discrete domain but was combined with the ‘multi/interdisciplinarity’ domain to create a more apposite domain of ‘training and expertise’. It was felt that the standards that could be created within both discrete domains covered very similar material, and so combining appeared sensible.
Output	None	This was not included as a domain because it was felt no standards could be formulated for it that either (a) were not so general that they were not already covered by generally accepted publishing standards or (b) were not too prescriptive about the kinds of outputs that empirical bioethics should aim for.
Research Ethics	None	This was not included as a domain because it was felt that the standards that were proposed under it simply replicated standards of practice that were already accepted and endorsed widely, and therefore having specific standards around research ethics for empirical bioethics was not needed.
Multi/ interdisciplinarity	None	This was not included as a discrete domain but was combinedwith the ‘training’ domain to create a more apposite domain of ‘training and expertise’. It was felt that the standards that could be created within both discrete domains covered very similar material, and so combining appeared sensible. The label was replaced with ‘expertise’ because it was considered overly prescriptive to require multi/Interdisciplinarity’. Rather, what was important is that all members of the team have appropriate training and expertise – and this was dictated by the research question and methods chosen.

For each domain and standard, participants were given the option of accepting the standard as it is, or rejecting it and providing reasons for the rejection. Participants were also invited to provide feedback on the domains and standards as a whole and had the opportunity to disagree with the list of domains (which had changed from the original round 2 list as a result of the round 5 discussion).

At the end of round 6b, all standards but 1 (standard 12, which required ‘explicit and robust ethical argument’) had reached 80% consensus (see Table 5).

**Table 5:** List of domains and standards and consensus reached during rounds 6a and 6b

Domain	Round 6a standards	% (*N* = 16)	Round 6b standards	% (*N* = 16)
Aims	1. Empirical bioethics research should address a normative issue that is oriented towards practice	94 (15/16)		
Aims	2. Empirical Bioethics research should integrate empirical methods with ethical arguments in order to address this normative issue	81 (13/16)		
Questions	3. Empirical bioethics researchers ought to be explicit about how the research question(s) asked address the normative issue identified in the aims	88 (14/16)		
Integration	4. The theoretical position on integration (i.e. the theoretical views on how the empirical and the normative are related) should be made clear and explicit	94 (15/16)		
Integration	5. The method of integration should be explained and justified, including details of what is integrated with what, how and by whom.	94 (15/16)		
Integration	6. There should be transparency, consistency and rigour in the execution and reporting of the integrating analysis	100 (16/16)		
Conduct of empirical work	7. Empirical bioethics research ought to attend to the rigorous implementation of empirical methods, and import accepted standards of conduct from appropriate research paradigms	94 (15/16)		
Conduct of empirical work	8. Empirical bioethics research should, if and where necessary, develop and amend empirical methods to facilitate collection of the data required to meet the aims of the research; but deviation from accepted disciplinary standards and practices ought to be acknowledged and justified	94 (15/16)		
Conduct of empirical work	9. Empirical bioethics research should reflect on and justify the appropriateness and fit of the chosen empirical methods in relation to (a) the normative aims and (b) the stated approach to integration	100 (16/16)		
Conduct of empirical work	10. Empirical bioethics research should consider and reflect on the implicit ethical and epistemological assumptions of the chosen empirical method	94 (15/16)		
Conduct of normative work	11. In empirical bioethics research there should be thorough delineation of the ethical issue(s), paying attention to, and locating them within, the relevant disciplinary literature	94 (15/16)		
Conduct of normative work	12. In empirical bioethics research there should be an explicit and robust ethical argument, where argument is understood as an explicit attempt to convince X to adopt position Y with the use of reasons	75 (12/16)	In empirical bioethics research there should be explicit and robust normative analysis. ‘Normative analysis’ includes attempts to justify position X to person Y with the use of ethical reasoning, providing suggestion for improvement to position X based on ethical reasoning, or attempts to break down and make explicit a complex normative issue in order to gain a better understanding of it	81 (13/16)
Training and expertise	13. The empirical bioethics researcher, or the research team as a whole, should possess competence in ethical inquiry, empirical inquiry and methods of integration	100 (16/16)		
Training and expertise	14. The empirical bioethics researcher(s) should have at least a basic knowledge of bioethics, and an understanding of whatever aspects of other disciplines or fields that are engaged with	88 (14/16)		
Training and expertise	15. Provision should be made for ensuring that any team members can acquire or enhance competence in empirical bioethics research	94 (15/16)		

#### Round 6b (post meeting) – Reformulation and second vote on standards.

In round 6b, the one outstanding standard was reworded by the meeting organizers to accommodate the feedback, and re-circulated in an online survey, with a covering letter containing all the feedback received on that standard and a rationale for the re-formulation. This re-formulation reached consensus (see Table 5).

In the remainder of this paper, we will focus on outlining and explaining each domain and standard that was agreed upon, based on the discussions participants had. We will also highlight areas that remain controversial despite consensus having been reached.

### Domain 1 - Aims

1. *Empirical bioethics research should address a normative issue that is oriented towards practice (94% consensus)*

The 94% consensus masks important conceptual clarification work undertaken in the consensus-building activities about the precise meaning of this standard. It was ultimately recognized that ‘a normative issue’ captures a point of ethical uncertainty and/or disagreement, concerning either a normatively defensible course of action or the normatively defensible use of a concept in practice. Thus, it was decided that a normative issue that aims to be settled in EB enquiry covers ethical uncertainty in two main areas:

i) When, in any given situation, it is uncertain whether course of action X or course of action Y ought to be pursued. For example, in obstetrics practice, whether doctors with religious views inconsistent with terminating pregnancies should be allowed to opt out of undertaking termination of pregnancy, or not.
ii) When, in any given situation, it is uncertain whether a concept relevant to an ethically defensible action should be understood in terms X or in terms Y. For example, in the context of dementia care, whether the concept of ‘respect for persons’ ought to be understood to foreground respect for a person’s expressed wishes as set down in advance (prior to the onset of dementia), or respect for their apparent wishes now.

Equally, much discussion took place about the precise meaning of the term ‘oriented towards practice’. First, it was recognized that the term ‘practice’ should be understood in broad terms. In this sense, it would incorporate practice as used in the traditional sense in bioethics to describe real-world actions in the settings of health care and biomedical research, but it would also include the formulation of policy- or rule-making to determine the form of real-world behaviours in these same areas.

Second, it was accepted that a defining feature of EB research was its aim to be connected to the real world in ways that can bring about ethically defensible changes to practice (understood in the broad sense articulated above). Notwithstanding this initial observation, participants also recognized that not each and every research activity in an EB project will be directly practicable – i.e. aiming to attempt to *bring about* such changes in the world. Therefore, the concept of ‘orientation’ was thought to best capture precisely how the practice focus of EB research ought to be understood.

2) *Empirical Bioethics research should integrate empirical methods with ethical argument* 4 *in order to address this normative issue (81% consensus)*

Disagreement in the early stages of consensus-building focused on which research activities were ‘in’ and which were ‘out’ of the gamut of EB research on account of their aims (not) being formulated explicitly in terms of addressing a normative issue through integrating empirical and normative work. It was agreed that this kind of standard was needed, and also that it would likely end up being controversial, because it amounts to a content-full criterion through which to determine whether or not an activity counts as EB. It was agreed that this standard needed to be formulated in broad terms, with an expansive understanding of ‘integration’, whilst simultaneously limiting empirical bioethics research to those studies that sought to address a normative issue.

It was also understood by all participants that this meant certain research activities would be excluded from counting as EB – but that this was unavoidable. What was important was ensuring that the standard was formulated clearly enough to make explicit what kinds of activities we are referring to, and therefore what kinds of activity ought to be held to the standards that are being formulated. The kinds of research activities that would be excluded based on this standard are:

i) Empirical research examining the form and nature of how ethical issues arise in practical situations (without any attempt to do more than characterize or describe these issues).
ii) Empirical research evaluating the implementation of interventions designed to bring about ethical practice, when these interventions are taken to have resolved all outstanding normative issues relating to the practice in question (i.e. where the normative issue underpinning the rationale for the intervention is not itself taken to be at issue in formulating the empirical research questions).
iii) Research drawing on empirical methods and ethical analysis to examine abstract philosophical problems that have no real-world correlates.
iv) Other empirical studies of moral behaviours or judgements that are not aiming to address normative ethical issues in practice.

### Domain 2 – Questions

3) *Empirical bioethics researchers ought to be explicit about how the research question(s) asked address the normative issue identified in the aims (88% consensus)*

There was relatively little disagreement about how the standard relating to research questions was formulated, despite the fact that this only achieved 88% consensus. Reflecting a widely recognized position in empirical research, the view that specific research questions ought to be connected directly to the overall aim of the research activity was not thought to be controversial, nor was it thought to be problematic to require researchers to be explicit about this connection.

Disagreement here tracked disagreement in the formulation of the second standard in the ‘aims’ domain, where there was uncertainty about whether one or more of these research questions would need to be formulated in normative terms. As with the ‘aims’ standard above, it was ultimately agreed that the research questions would only meet the requisite standard for EB research if they were formulated in such a way as to address the normative issue in ways that were oriented towards practice. Thus, it would be expected that one or more research questions would need to be a normative (ought) question, and the connection to a particular field of practice would need to be articulated within the normative question.

### Domain 3 – Integration

The three standards within the ‘integration’ domain refer to the integration of empirical data^5^ and normative analysis, comprising some kind of normative reasoning process in which the empirical data are included.

When working towards formulating these standards there was discussion and disagreement about the meaning of ‘integration’ and what the concept implies. Some felt that integration does not have to mean that the empirical and the normative are integrated into one kind of knowledge entity, and that empirical data and normative reasoning remain separate and essentially different entities with empirical data supporting empirical premises in normative argument. Others had a different perspective, feeling that the requirement to engage with the concept of integration was the construct of an artificial philosophical separation, and that the empirical and the normative are already inseparably entangled. Some felt that all ‘integration’ can really mean is that empirical data are used to help reach normative goals, whereas others felt that empirical data itself has normative status independent of any argument.

The shared agreement was that standards in this domain should not be prescriptive about what integration means, but should focus on ensuring that formal criteria of transparency and justification are met. Participants acknowledged that transparency requires articulation of the meta-ethical and epistemological basis of one’s position on integration, but also that there can be reasonable disagreement on this issue; importantly, that the appropriateness of any position on integration has to be assessed in the context of the aims of the research. Given this, participants felt it was essential that EB researchers clarify how they think about integration and how they undertake it, justifying their chosen approach in relation to the aims of the research.

4) *The theoretical position on integration (*i.e. *the theoretical views on how the empirical and the normative are related) should be made clear and explicit (94% consensus)*

The only reason this standard did not reach 100% consensus was that one participant felt it could and should be combined with standard 5. With respect to this standard, the EB researcher should clarify his or her theoretical viewpoint on integration. This would require, for example, an articulation of the meta-ethical and epistemological positions that allow normative knowledge claims to be produced. For example, if one adopted a hermaneutical/dialogical approach to integration, one would need to provide (or refer to) an account of how this approach enables us to make justified knowledge claims about right and wrong based on articulation of the justificatory power of dialogue and consensus.

The main point of contestation when this standard was being discussed and formulated was a concern about the extent to which these positions had to be justified and defended, or simply referred to clearly. The consensus position very intentionally says ‘make clear and explicit’ rather than ‘justify or defend’ because it was felt, overall, that it would be unreasonably burdensome to expect researchers to defend all meta-ethical and epistemologial commitments and assumptions in every paper. Rather, it was felt to be sufficient to make clear what they are and provide appropriate citations according to standard academic convention.

5) *The method of integration should be explained and justified, including details of what is integrated with what, how and by whom (94% consensus)*

The only reason this standard did not reach 100% consensus was that one participant felt it could and should be combined with standard 4. The primary reason it seemed nonetheless appropriate to maintain a distinction between them is that standard 4 requires certain positions and assumptions to be made clear. Standard 5, however, requires clear articulation of the implications of those positions in the context of this specific project, and explanation of how the combination of meta-ethics, epistemology and method form a methodology capable of meeting the project’s stated aims. As such, standard 5 operates at a higher level of specificity than standard 4, and requires justification as opposed to mere articulation. This seems appropriate, because whilst it is arguably unreasonable to require a researcher to fully justify a longstanding meta-ethical and/or epistemological position in order to present their research, it seems nonetheless essential that the researcher explains how that position has an impact on their methodology for a specific project and therefore affects the knowledge claims that are produced, and why the decisions made are justified given the aims of the research.

6) *There should be transparency, consistency and rigor in the execution and reporting of the integrating analysis (100% consensus)*

This standard requires a transparent description of the analytic process that leads to the normative conclusion(s) – essentially a narrative that makes clear the process of reasoning that led to these specific normative conclusions being drawn. Meeting this standard requires a further level of specificity as it asks the researcher to articulate clearly how the methodology was applied to create a reasoning process that can be followed and understood.

It is important to note that during discussions about all three of the integration standards, some participants repeatedly pointed towards the fact that the term ‘integration’ seems to presuppose a clear distinction between ‘descriptive’ and ‘normative’ (or prescriptive) components of ethics research, such that processes through which they are combined (integrated) are required. These participants wanted to remind us that some practitioners of EB research do not accept this distinction. They were, however, generally willing to support these standards of integration, and this seems justifiable given that one way to meet standard 4 would be to articulate a meta-ethical/epistemological position that either makes integration unnecessary or provides an alternative account of the relationship between facts and values.

### Domain 4 – Conduct of empirical work

7) *Empirical bioethics research ought to attend to the rigorous implementation of empirical methods, and import accepted standards of conduct from appropriate research paradigms (94% consensus)*

This standard reflects and attends to concerns raised, for example by Hurst [3], that some EB work uses methods and approaches from discrete disciplines but does not apply the standards of rigour that those disciplines require. It stands to reason that if one is importing a method of either empirical or conceptual analysis, and relying on it to inform some part of the research process, then one cannot use interdisciplinary as an excuse for poor execution of that method. One issue that arises with this standard is the need to distinguish between conduct and reporting. It is a well-known problem for interdisciplinary researchers that even assuming all aspects of the research were conducted according to this standard, when it comes to reporting the work there is often insufficient space to demonstrate and report that rigor in full. As such, one may end up focusing on demonstrating rigor in one aspect of the work (dictated by the disciplinary focus of the target journal) at the expense of another. The point here is that whilst this standard is easy to formulate, and relatively straightforward to implement, we recognize that it is challenging to demonstrate and report this rigor given current publication norms.

8) *Empirical bioethics research should, if and where necessary, develop and amend empirical methods to facilitate collection of the data required to meet the aims of the research; but deviation from accepted disciplinary standards and practices ought to be acknowledged and justified (94% consensus)*

This standard functions to signal, and acknowledge, the often necessary innovation and adaptation required in EB. Empirical methods that are borrowed from established disciplines may not be entirely fit for purpose, and may need developing to enable a specific project to meet its aims. One good example of this is making qualitative research encounters more interrogative, with the researcher challenging the participant and offering counterfactual cases to explore the values that might lie behind an ethical stance articulated by the participant in an interview. This statement is important to consider in combination with standard 7. Whilst methods must be executed with appropriate disciplinary rigor, when a method is not fit for purpose in a specific research project it is necessary to amend it – but such amendment must be acknowledged and justified.

9) *Empirical bioethics research should reflect on and justify the appropriateness and fit of the chosen empirical methods in relation to (a) the normative aims (b) the stated approach to integration (100% consensus)*

This standard refers to the requirement to harmonize the empirical methods used with the aims of the research, the process of integration and the analysis – and it is implicit that this includes the moral epistemology adopted. This is perhaps one of the most difficult things to grasp for empirical bioethics researchers. The fact that different approaches to empirical research are founded upon different epistemological commitments is something to which some philosophical approaches to empirical bioethics have failed to be attentive [27, 28]. To not recognize this, and to not harmonize all stages of the research, is to fail to see the research process as a coherent whole and rather views it as a patchwork where different methods and epistemologies can be swapped in and out unproblematically. For example, if one intends to draw generalizable normative conclusions through the combination of empirical and ethical analysis, one need to consider whether, and why, one might need to use empirical research methods that are compatible with making generalizable knowledge claims.

10) *Empirical bioethics research should consider and reflect on the implicit ethical and epistemological assumptions of the chosen empirical method (94% consensus)*

This standard is a pre-requisite for being able to meet standard 9, and refers to the requirement to be aware of the fact that empirical methods themselves are not value free tools that are capable of producing objective empirical knowledge claims (see also Singh, 2017). Different empirical methods prioritize different kinds of knowledge production, for example; focus groups are designed to capture a group view; in-depth interviews are designed to capture personal accounts; large population surveys are designed to generate generalizable knowledge claims about a population. Given this, choosing a particular empirical method signals a prioritization of certain kinds of knowledge claim in terms of both (a) what kind of empirical knowledge is most valuable and (b) what kind of knowledge claim is most appropriate to meet the aims of the project. Bearing in mind that the chosen methods will tend to place limits or expectations on, for example: the number of people that can be included as participants; the extent to which individual voices are listened to; or the kinds of people who can participate, the choice of empirical method signals who and what is important to the researcher, and why. It is important, therefore, to be able to consider to what extent the implications of choices made about the empirical method are compatible with the normative aims of the research.

### Domain 5 – Conduct of normative work

11) *In empirical bioethics research there should be thorough delineation of the ethical issue(s), paying attention to, and locating them within, the relevant disciplinary literature (94% consensus)*

This standard reflects the view that regardless of how the normative analysis is undertaken, EB work must make clear the ethical issues it is concerned with and take pains to locate those issues within the existing literature. This is important, given that it is possible to identify some (putatively) EB work that either (a) takes for granted and assumes what the ethical issues are, or (b) fails to acknowledge that much ‘mono-disciplinary’ work on the normative issues may have already been conducted (or assumes that work is no longer relevant now that empirical work has been or is being done). This formulation allows for work that begins from a position of having identified the ethical issues, or that seeks to discover them along the way. What it does is state that, at some point appropriate to the precise research process being used: (a) the ethical issues the work is concerned with must be clearly articulated; (b) an explanation must be provided about why they are *ethical* issues, and; (c) there must be engagement with relevant disciplinary literature that has dealt with the same or relevantly similar issues.

12) *In empirical bioethics research there should be explicit and robust normative analysis. ‘Normative analysis’ includes attempts to justify position X to person Y with the use of ethical reasoning, providing suggestion for improvement to position X based on ethical reasoning, or attempts to break down and make explicit a complex normative issue in order to gain a better understanding of it (81% consensus)*

This standard was the most controversial amongst the group, and only reached consensus after re-formulation and then only at 81% (the lowest possible). This was also the most controversial standard at the meeting itself, taking up in excess of 90 min of the round 5 discussion. The initial framing was considered too narrow in its use and definition of the term ‘ethical argument’, and discussion focused on ways of making the standard less restrictive in terms of the normative work it supported. The main issues are captured well in the following comment offered during round 6a:

*“Ethical arguments” is too narrow to capture other methods that classify [*sic*] as “normative analysis”. Furthermore, I do not see how this broader term “normative analysis” would open the door for an inflationary broadening that risks losing the genuine normative elements that we would like to see in our definition of empirical bioethics. I would therefore prefer the broader term “normative analysis”.*

In the reformulation, the term ‘normative analysis’ was adopted, and examples provided that encompass different kinds of normative work that do not focus on ethical argument that attempts to convince others of the argument’s conclusion, but also includes (a) normative work that develops new insights that could broaden one’s moral horizon (stimulating reflection and dialogue, and presented as questions for investigation rather than arguments intended to convince), or (b) elucidating ethical problems by showing that currently accepted ways of resolving them do not do justice to all relevant aspects of the problem.

This standard remains, perhaps, the most controversial. Some participants felt that any attempt to provide insight, elucidate or make suggestions must include at least an implicit attempt to convince the reader that such claims ought to be taken seriously, and in doing that one is necessarily making an argument that one’s own point of view ought to be accepted. Further, it might be claimed that if no such attempt is being made, there is nothing normative about the work and it is therefore merely describing possible positions and options. Conversely, others felt that ‘normative analysis’ can aim to provide insight into an ethical problem by showing various possible issues and perspectives, stimulating a broadening of one’s moral horizon – without engaging in *argument* of any kind. The normative claim in such work would be that these broader perspectives should be taken into account, without providing an argument as to why one of them would be the best (which would contradict the aim of opening up new ways of envisaging a moral problem). The putative difference between normative argument and normative analysis is, therefore, that the former attempts to convince an interlocutor to adopt a specific position, whereas the latter can offer a range of options and perspectives that can broaden existing positions and arguments - thus providing a basis for further argument - without claiming to provide a decisive conclusion.

Some participants felt strongly that the term ‘argument’ excluded anything that was not an attempt to convince, whereas the term ‘analysis’ includes both attempts to convince and other kinds of normative work. Other participants felt just as strongly that unless there was some attempt to convince – to make a ‘should’ or an ‘ought’ claim, there is nothing normative going on, and additionally that any attempt to articulate perspectives that decision makers ‘should’ take into account *must* be making an argument that people should act in certain way.

Overall, however, the consensus group felt that the entire spectrum of normative work ought to be accommodated, and that the language of ‘analysis’ was more unproblematically inclusive than the language of ‘argument’. Even so, this was still the standard with the lowest level of consensus, and it remains at issue.

### Domain 6 – Training and expertise

13) *The empirical bioethics researcher, or the research team as a whole, should possess competence in ethical inquiry, empirical inquiry and methods of integration (100% consensus)*

In line with the previous domains and standards, this standard requires that core competences for conducting EB research must be possessed by, or accessible to, the conducting researcher or research team. While in recent years many researchers have acquired competency in normative and empirical disciplines, as well as the competence to integrate normative and empirical analyses, there was acknowledgement that in many, if not most, cases the necessary competences will need to be contributed by different researchers. However it is provided or accessed, it was considered essential by all that such competencies are present in the research team.

14) *The empirical bioethics researcher(s) should have at least a basic knowledge of bioethics, and an understanding of whatever aspects of other disciplines or fields that are engaged with (88% consensus)*

On one hand, this standard emphasizes that in EB knowledge in bioethics is the central prerequisite for any competent research. On the other hand, the standard acknowledges that knowledge and/or skills related to other normative or empirical disciplines are important for EB research. The relatively low figure for consensus on this standard, compared to the 100% consensus reached for standard 13, may be explained by the discussion about what participants understand by “basic knowledge of bioethics” given the breadth of the field. For example, the group recognized they could not have agreed on a minimal set of theories, concepts or topics which comprise a threshold of basic bioethics knowledge - and in the absence of this a standard might seem quite empty. Additionally, the phrase “understanding of whatever aspects of other disciplines or fields” had to be formulated in very broad terms so it would cover the broad range of knowledge, methods and skills that might be needed for a specific project. The formulation of this standard attempts to make clear that, whatever one is doing, one ought to consider whether, and be able to show that, one has sufficient understanding and basic knowledge to do it.

15) *Provision should be made for ensuring that any team members can acquire or enhance competence in empirical bioethics research (93% consensus)*

This standard is clearly connected to standards 13 and 14, but is differentiated by the fact that it requires not only that those competences are delivered as part of an EB research project, but also that those participating in the respective research can acquire the relevant knowledge and skills during the project. While this standard, and the others in this domain, may not be relevant in evaluating the EB research paper for publication, they are highly relevant for the evaluation of EB research proposals involving junior researchers from different disciplines who wish to become competent EB researchers.

### Final remarks

Having outlined the 15 standards for EB research on which we were able to reach consensus, it is important to consider how they should be implemented and understood. There are a few points we would like to make in relation to this.

First, it is clear that these standards cover a range of research phases, and not all of the standards will or should be applicable to each phase. The most basic distinctions to make are between identifying and designing an EB project, planning, carrying out the project, and reporting it. To make clear to which research phases we anticipate these standards applying, we present Table 6, which indicates the standard, the research phase, and how we see the standard applying to that phase.

**Table 6:** Detailing how each standard can be met at various stages of the research process

Standard	Identifying (if a project does not meet these standards, it is not the kind of project to which the rest of the standards apply)	Planning (required for evaluation of a project plan for, e.g. funding)	Carrying out	Reporting (these standards should be met as far as possible, accepting space constraints in journals. Online supplementary material might be considered)
1. Empirical bioethics research should address a normative issue that is oriented towards practice	The aims of the project must make explicit the normative issue being addressed and the practical end to which the research is directed.	The extent to which the research aims to address a normative issue oriented towards practice should be explicitly stated.	N/A	The extent to which the research aimed to address a normative issue oriented towards practice should be explicitly stated.
2. Empirical Bioethics research should integrate empirical methods with ethical arguments in order to address this normative issue	The research should not simply be an empirical project being carried out alongside an ethics project, but involves some kind of integration where each mutually informs the other.	The method of integration ought to be considered at the planning stage.	N/A	Any integration should be clearly articulated so that it is intelligible to the reader.
3. Empirical bioethics researchers ought to be explicit about how the research question(s) asked address the normative issue identified in the aims	N/A	It should be explicitly articulated in advance how the normative aims of the project can be met by answering the specified research questions.	N/A	It should be clearly articulated how the normative aims of the project were met and the research questions answered.
4. The theoretical position on integration (i.e. the theoretical views on how the empirical and the normative are related) should be made clear and explicit	N/A	The research plan should clearly articulate the meta-ethical and epistemological positions that allow knowledge claims to be made on the basis of the planned research.	N/A	The report should clearly articulate the meta-ethical and epistemological positions that allowed knowledge claims to be made.
5. The method of integration should be explained and justified, including details of what is integrated with what, how and by whom.	N/A	The research plan should include a clear account of how the integrated analysis will be undertaken, such that it can be understood by a person unfamiliar with the project.	N/A	The report should include a clear account of how the integrated analysis was undertaken, such that it can be understood by the reader.
6. There should be transparency, consistency and rigour in the execution and reporting of the integrating analysis	N/A	N/A	The project should be carried out according to the plan, as far as possible, with deviations recorded and justified.	The report should be transparent in explication of the analytic processes.
7. Empirical bioethics research ought to attend to the rigorous implementation of empirical methods, and import accepted standards of conduct from appropriate research paradigms	N/A	The plan ought to consider, provide an account of, and justify, how empirical methods will be appropriately used – appealing to disciplinary standards as appropriate.	Empirical methods ought to be used according to appropriate standards of rigor.	The report ought to provide an account of how empirical methods were rigorously implemented.
8. Empirical bioethics research should, if and where necessary, develop and amend empirical methods to facilitate collection of the data required to meet the aims of the research; but deviation from accepted disciplinary standards and practices ought to be acknowledged and justified	N/A	The plan ought to consider, provide an account of, and justify, how empirical methods might be appropriately amended.	Amendments to (or deviations from) standard approaches to collecting empirical data ought to be made when they are required.	The report ought to provide an explanation and explicit justification of any amendments made to standard empirical methods.
9. Empirical bioethics research should reflect on and justify the appropriateness and fit of the chosen empirical methods in relation to (a) the normative aims (b) the stated approach to integration	N/A	The research plan should consider and explain how the chosen empirical methods are compatible with the normative aims and the planned approach to integration.	N/A	The report should provide an account of the compatibility of the chosen empirical methods with the normative aims and the approach to integration taken.
10. Empirical bioethics research should consider and reflect on the implicit ethical and epistemological assumptions of the chosen empirical method	N/A	The research plan should acknowledge the ethical and epistemological assumptions behind the project’s method(s), and consider how this will impact on the knowledge claims that can be made.	N/A	The report should acknowledge the ethical and epistemological assumptions behind the project’s empirical method(s), and consider the ways in which these might place limitations on the conclusions that have been drawn.
11. In empirical bioethics research, there should be thorough delineation of the ethical issue(s), paying attention to, and locating them within, the relevant disciplinary literature	N/A	The research plan should clearly articulate the ethical issues that are being investigated, ensuring that due attention is paid to the range of literatures on the topic that may exist.	A literature search ought to be conducted, which is sufficiently rigorous to capture all relevant material (within reason).	The report should clearly and explicitly articulate how the project has engaged with the ethical issues that drove it, situating its own treatment of them within the wider literature from relevant disciplines.
12. In empirical bioethics research, there should be explicit and robust normative analysis. ‘Normative analysis’ includes attempts to justify position X to person Y with the use of ethical reasoning, providing suggestion for improvement to position X based on ethical reasoning, or attempts to break down and make explicit a complex normative issue in order to gain a better understanding of it	N/A	The research plan should articulate what kind of normative analysis will be undertaken and how it will be done.	A normative analysis should be carried out.	The report should contain a clear explication of the normative analysis, including its process and its conclusions.
13. The empirical bioethics researcher, or the research team as a whole, should possess competence in ethical inquiry, empirical inquiry and methods of integration	N/A	The research plan should consider the competencies required to undertake the project and ensure that these competencies are available and accessible.	The project should be carried out by researchers in possession of the required competencies.	N/A
14. The empirical bioethics researcher(s) should have at least a basic knowledge of bioethics, and an understanding of whatever aspects of other disciplines or fields that are engaged with	N/A	Knowledge and understanding of bioethics and other relevant disciplines should be demonstrated in the research plan.	The researcher or research team should possess sufficient knowledge and understanding of bioethics and other relevant disciplines to be capable of carrying out the project.	N/A
15. Provision should be made for ensuring that any team members can acquire or enhance competence in empirical bioethics research	N/A	The research plan should include training plan, as appropriate, to ensure that the researcher or research team has the appropriate competencies.	N/A	N/A

Second, in outlining standards of practice we are making an explicit normative point about what a certain kind of work should look like, and must therefore consider the implications for a piece of work that does not meet them. In setting out standards of practice we are outlining criteria that might be used to both identify research as EB research and appraise the quality of EB research. The majority of the standards we have articulated, however, are formal rather than contently – meaning that they set out requirements for features of the research process that need to be attended to rather than stipulating a priori how they ought to be attended to. In this sense, these standards can be understood as minimal criteria, similar to those seen in the literature on quality appraisal of qualitative research [29, 30], which outline what researchers need to do in order to demonstrate they are giving consideration to key features of the research process and enable a judgement to be made about its quality. What they cannot do is stipulate, a priori*,* whether the decisions made in relation to these features of research, or the discussion of them that is provided, is good quality. That remains a matter of judgement.

The exceptions to this are standards 1 and 2, which, as Table 6 shows, we see as standards that will determine whether a particular piece of research is the kind of research to which the rest of the standards ought to apply. These standards do make contently claims, but this seems essential given what they aim to do.

It is, of course, important to consider this point alongside the limitations (discussed above) imposed by a process that included only a limited group of European researchers. The consensus on standards 1 and 2 in particular may well be a product of a particularly European perspective, and is certainly a product of the particular group of individuals who took part.

Similarly, further discussion and debate may be needed to consider whether we were correct to focus our consensus on a framing of empirical bioethics that is limited to the integration of social scientific and ethical analysis. Alternative perspectives might want to include the integration of data from the life sciences, and this would be legitimate enterprise. Although this has not featured significantly in the literature to date, one notable exception is a recent paper by Mertz and Schildmann [31], who have suggested that the methodological challenges therein may not be dissimilar to those encountered when we focus on social scientific data.

We appreciate that our standards could lead to some research practices being excluded as EB, and others being included when those who pursue it might not want them to be. We hope that this exercise is not perceived as an attempt to draw boundaries, but rather as an attempt by a developing community of practice [22] to say ‘if we want to engage in this kind of research practice, which has these kinds of aims, these are the standards to which we agree we should hold ourselves’. The act of labelling this research practice ‘Empirical Bioethics’ might be interpreted as a territorial act and an appropriation of the term. We see it, rather, as a necessary step in achieving clarity. Whilst others are free to disagree with what we have done, this exercise will help to clarify precisely what they are disagreeing with. A useful exercise that might be undertaken next is to gather examples of EB research that either demonstrate adherence to, or challenge, the standards outlined here; focusing on examples that illustrate good practice according to, or in spite of not adhering to, these standards.

That said, we can also view this consensus statement as a step towards fully engaging the wider community of EB researchers in a discussion that needs to continue. Through articulating these standards we outline a position on empirical bioethics that encourages responses, and through those responses we will be able to identify points of agreement and contestation that will drive the conversation forward. In that vein, we would encourage researchers, funders and journals to engage with what we have proposed, and respond to us, so that our community of practice of EB research can develop and evolve further.

## Abbreviation

EB: Empirical Bioethics

## Footnotes

1 Ontological questions are, broadly speaking, questions about the nature of substance and reality. Moral ontology is the study of the nature and substance of morality (e.g. are moral values objectively real and independent of the person making the judgement?).

2 Epistemological questions are, broadly speaking, questions about the nature of knowledge. Moral epistemology is the study of the nature of moral knowledge (e, g, how can we obtain knowledge about what is right and wrong).

3 A meta-ethical position is, broadly speaking; a set of beliefs or commitments about the nature of moral claims and how one can have knowledge about right and wrong.

4 It was noted during the writing up stage that this framing of ‘ethical arguments’ seems at odds with the framing of ‘ethical analysis’ articulated, and much debated, in standard 12 (below). Whilst it is important, for the integrity of the consensus process, to maintain the wording of this standard that was agreed, it may be reasonable to assume a wider reading of ‘ethical argument’ here that is consistent with the use of ‘normative analysis’ in standard 12.

5 Participants acknowledged that ‘empirical data’ can mean different things. For example, biological parameters related to experiencing pain, answers to questionnaires on normative attitudes towards a particular issue, but also dialogue with stakeholders in the practice under study. We did, however, focus on the use of social scientific data, rather than, for example, data from the natural sciences.
